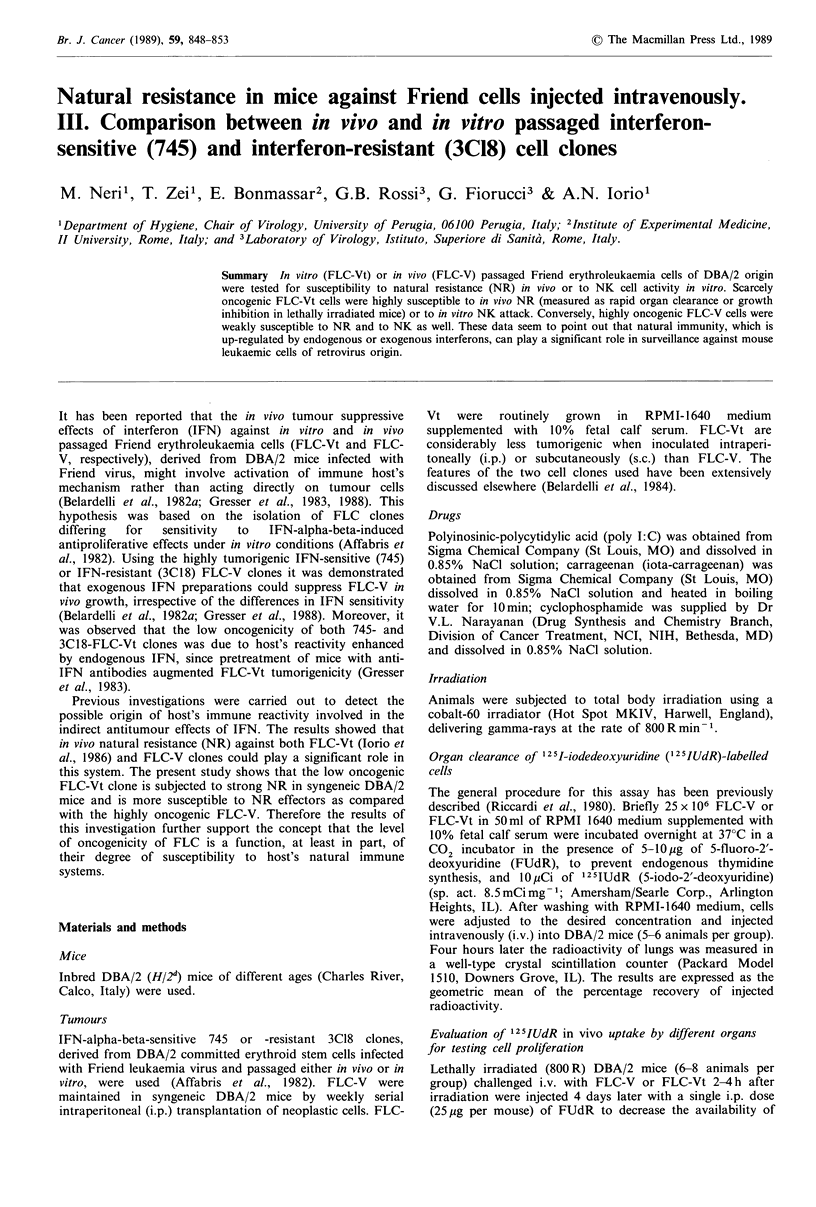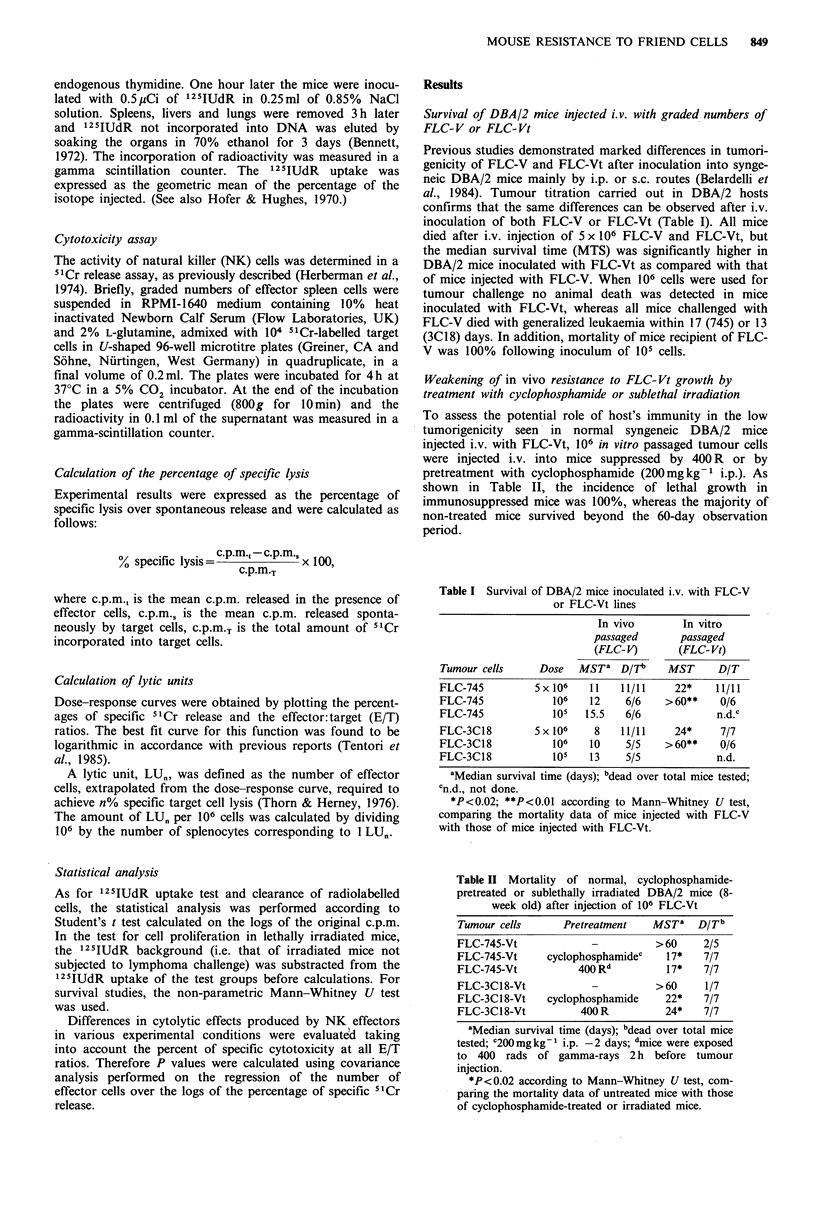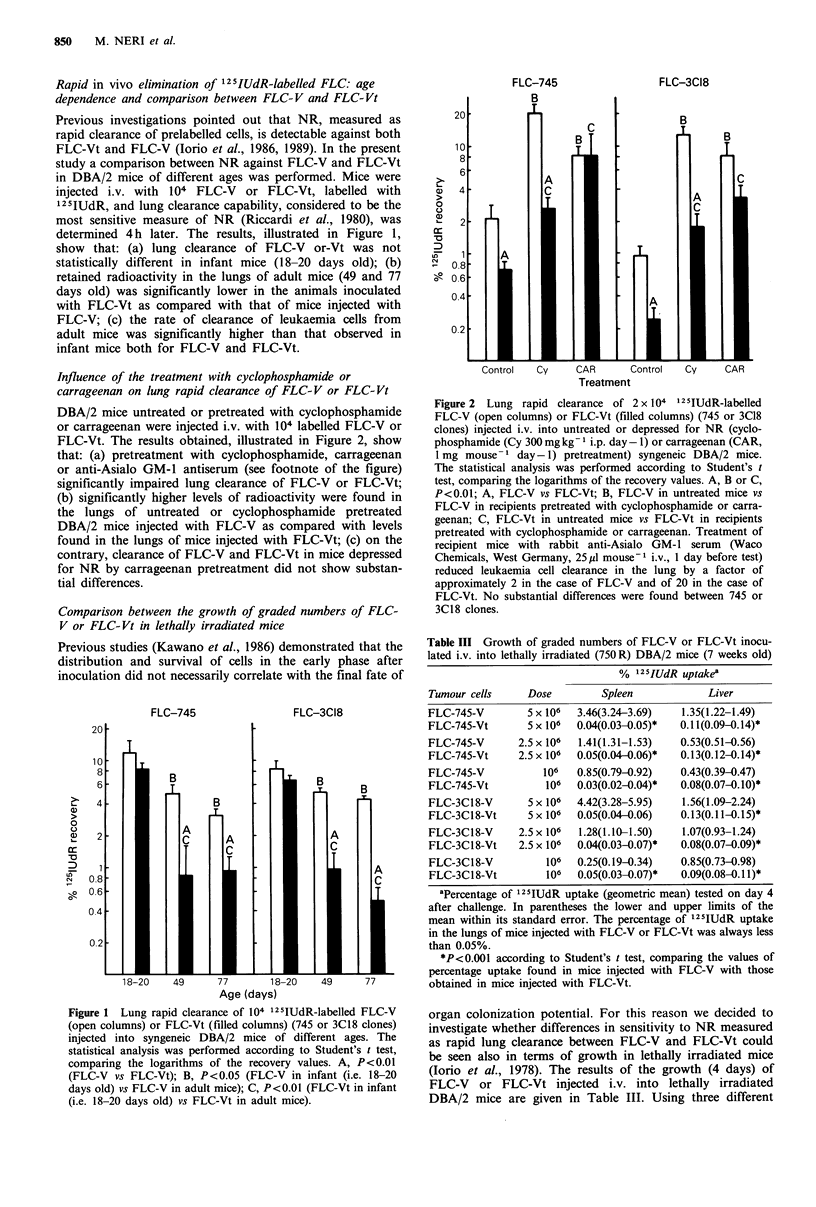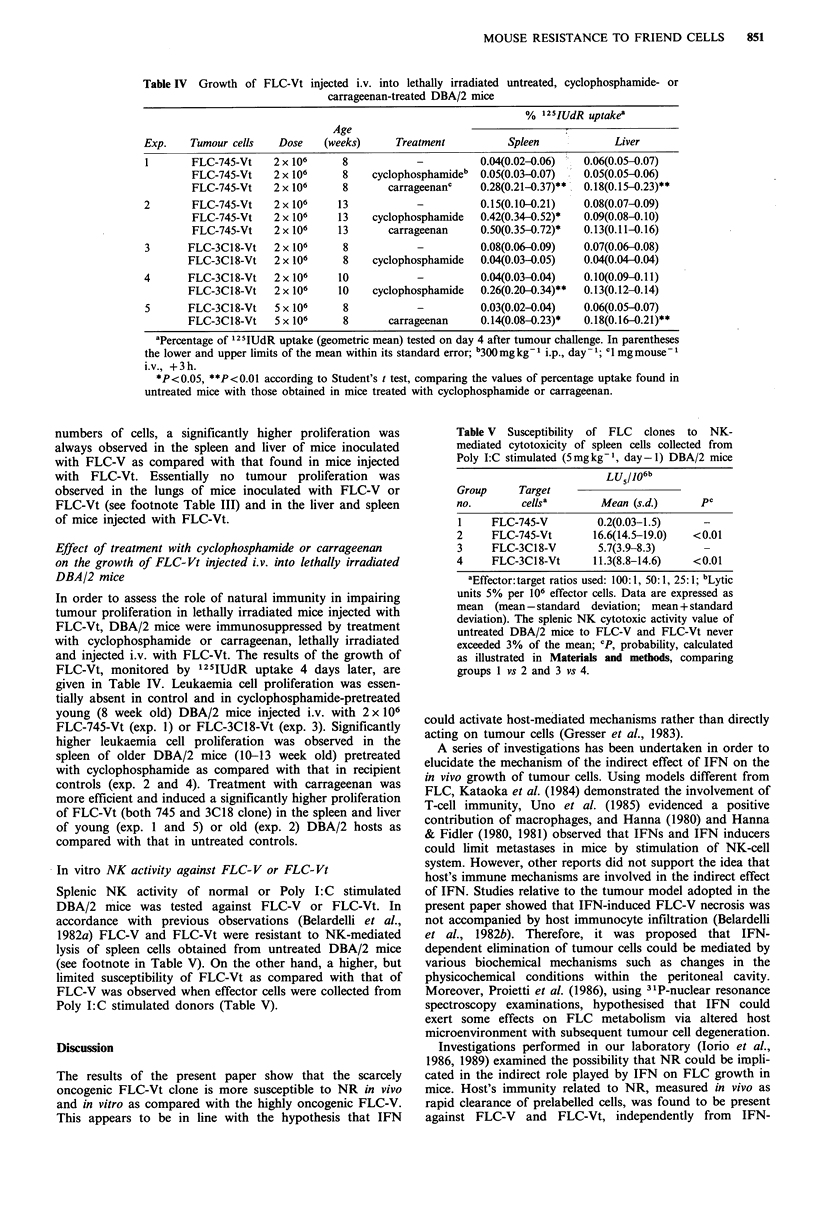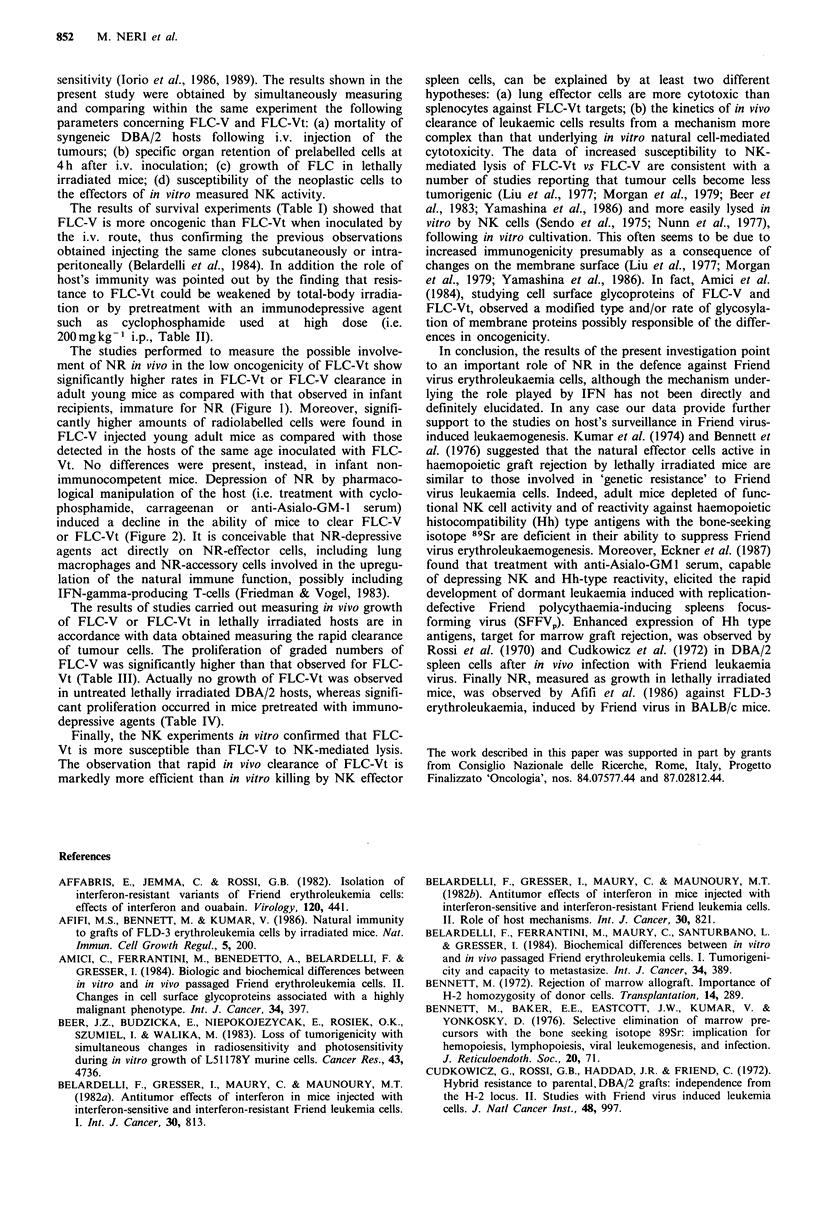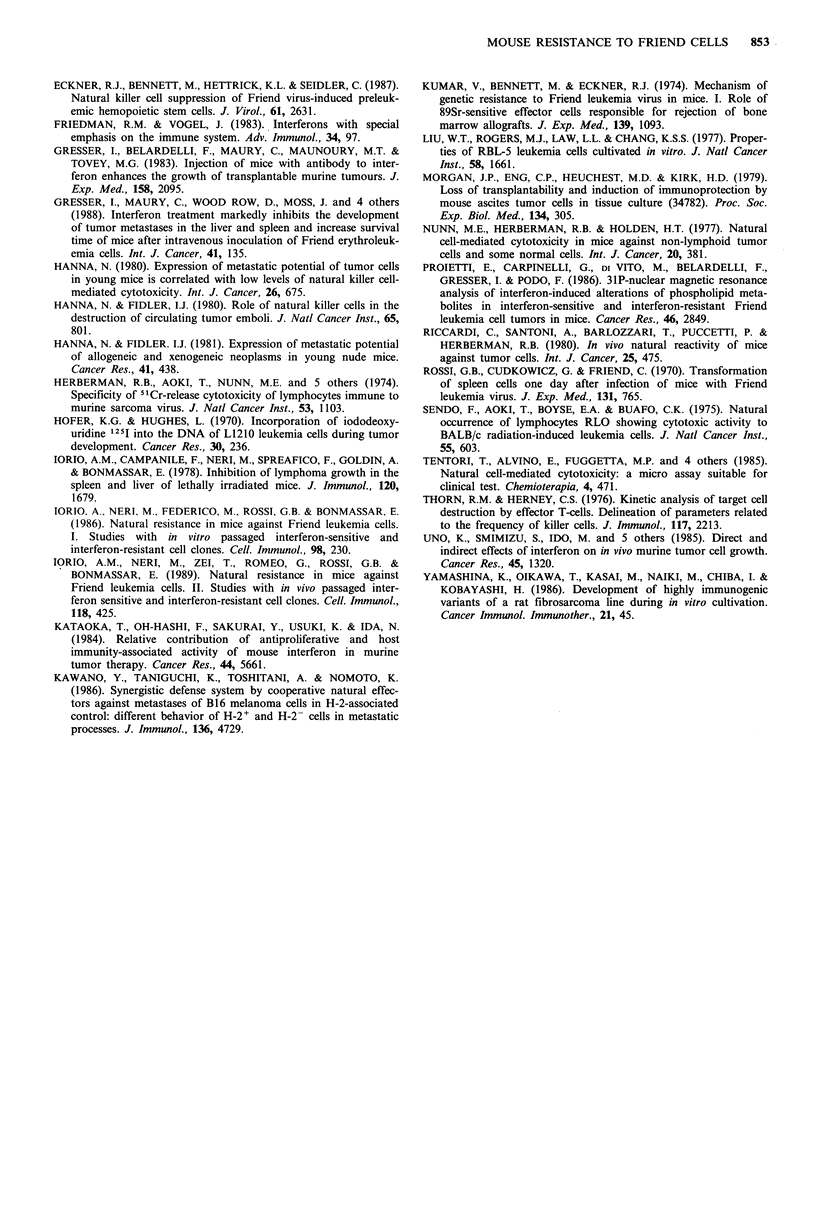# Natural resistance in mice against Friend cells injected intravenously. III. Comparison between in vivo and in vitro passaged interferon-sensitive (745) and interferon-resistant (3Cl8) cell clones.

**DOI:** 10.1038/bjc.1989.181

**Published:** 1989-06

**Authors:** M. Neri, T. Zei, E. Bonmassar, G. B. Rossi, G. Fiorucci, A. N. Iorio

**Affiliations:** Department of Hygiene, University of Perugia, Italy.

## Abstract

In vitro (FLC-Vt) or in vivo (FLC-V) passaged Friend erythroleukaemia cells of DBA/2 origin were tested for susceptibility to natural resistance (NR) in vivo or to NK cell activity in vitro. Scarcely oncogenic FLC-Vt cells were highly susceptible to in vivo NR (measured as rapid organ clearance or growth inhibition in lethally irradiated mice) or to in vitro NK attack. Conversely, highly oncogenic FLC-V cells were weakly susceptible to NR and to NK as well. These data seem to point out that natural immunity, which is up-regulated by endogenous or exogenous interferons, can play a significant role in surveillance against mouse leukaemic cells of retrovirus origin.


*Br. J. Cancer* (1989), **59**, 848–853 © The Macmillan Press Ltd., 1989

### Natural resistance in mice against Friend cells injected intravenously. III. Comparison between *in vivo* and *in vitro* passaged interferon-sensitive (745) and interferon-resistant (3C18) cell clones

M. Neri[1], T. Zei[1], E. Bonmassar[2], G.B. Rossi[3], G. Fiorucci[3] & A.N. Iorio[1]

[1]*Department of Hygiene, Chair of Virology, University of Perugia, 06100 Perugia, Italy;* [2]*Institute of Experimental Medicine, II University, Rome, Italy; and* [3]*Laboratory of Virology, Istituto, Superiore di Sanità, Rome, Italy.*

**Summary** *In vitro* (FLC-Vt) or *in vivo* (FLC-V) passaged Friend erythroleukaemia cells of DBA/2 origin were tested for susceptibility to natural resistance (NR) *in vivo* or to NK cell activity *in vitro*. Scarcely oncogenic FLC-Vt cells were highly susceptible to *in vivo* NR (measured as rapid organ clearance or growth inhibition in lethally irradiated mice) or to *in vitro* NK attack. Conversely, highly oncogenic FLC-V cells were weakly susceptible to NR and to NK as well. These data seem to point out that natural immunity, which is up-regulated by endogenous or exogenous interferons, can play a significant role in surveillance against mouse leukaemic cells of retrovirus origin.

It has been reported that the *in vivo* tumour suppressive effects of interferon (IFN) against *in vitro* and *in vivo* passaged Friend erythroleukaemia cells (FLC-Vt and FLC-V, respectively), derived from DBA/2 mice infected with Friend virus, might involve activation of immune host's mechanism rather than acting directly on tumour cells (Belardelli *et al.*, 1982*a*; Gresser *et al.*, 1983, 1988). This hypothesis was based on the isolation of FLC clones differing for sensitivity to IFN-alpha-beta-induced antiproliferative effects under *in vitro* conditions (Affabris *et al.*, 1982). Using the highly tumorigenic IFN-sensitive (745) or IFN-resistant (3C18) FLC-V clones it was demonstrated that exogenous IFN preparations could suppress FLC-V *in vivo* growth, irrespective of the differences in IFN sensitivity (Belardelli *et al.*, 1982*a*; Gresser *et al.*, 1988). Moreover, it was observed that the low oncogenicity of both 745- and 3C18-FLC-Vt clones was due to host's reactivity enhanced by endogenous IFN, since pretreatment of mice with anti-IFN antibodies augmented FLC-Vt tumorigenicity (Gresser *et al.*, 1983).

Previous investigations were carried out to detect the possible origin of host's immune reactivity involved in the indirect antitumour effects of IFN. The results showed that *in vivo* natural resistance (NR) against both FLC-Vt (Iorio *et al.*, 1986) and FLC-V clones could play a significant role in this system. The present study shows that the low oncogenic FLC-Vt clone is subjected to strong NR in syngeneic DBA/2 mice and is more susceptible to NR effectors as compared with the highly oncogenic FLC-V. Therefore the results of this investigation further support the concept that the level of oncogenicity of FLC is a function, at least in part, of their degree of susceptibility to host's natural immune systems.

### Materials and methods

### *Mice*

Inbred DBA/2 ($H/2^d$) mice of different ages (Charles River, Calco, Italy) were used.

### *Tumours*

IFN-alpha-beta-sensitive 745 or -resistant 3C18 clones, derived from DBA/2 committed erythroid stem cells infected with Friend leukaemia virus and passaged either *in vivo* or *in vitro*, were used (Affabris *et al.*, 1982). FLC-V were maintained in syngeneic DBA/2 mice by weekly serial intraperitoneal (i.p.) transplantation of neoplastic cells. FLC-Vt were routinely grown in RPMI-1640 medium supplemented with 10% fetal calf serum. FLC-Vt are considerably less tumorigenic when inoculated intraperitoneally (i.p.) or subcutaneously (s.c.) than FLC-V. The features of the two cell clones used have been extensively discussed elsewhere (Belardelli *et al.*, 1984).

### *Drugs*

Polyinosinic-polycytidylic acid (poly I:C) was obtained from Sigma Chemical Company (St Louis, MO) and dissolved in 0.85% NaCl solution; carrageenan (iota-carrageenan) was obtained from Sigma Chemical Company (St Louis, MO) dissolved in 0.85% NaCl solution and heated in boiling water for 10 min; cyclophosphamide was supplied by Dr V.L. Narayanan (Drug Synthesis and Chemistry Branch, Division of Cancer Treatment, NCI, NIH, Bethesda, MD) and dissolved in 0.85% NaCl solution.

### *Irradiation*

Animals were subjected to total body irradiation using a cobalt-60 irradiator (Hot Spot MKIV, Harwell, England), delivering gamma-rays at the rate of $800\,R\,min^{-1}$.

### *Organ clearance of $^{125}I$-iodedeoxyuridine ($^{125}IUdR$)-labelled cells*

The general procedure for this assay has been previously described (Riccardi *et al.*, 1980). Briefly $25 \times 10^6$ FLC-V or FLC-Vt in 50 ml of RPMI 1640 medium supplemented with 10% fetal calf serum were incubated overnight at 37°C in a $CO_2$ incubator in the presence of 5–10 $\mu$g of 5-fluoro-2′-deoxyuridine (FUdR), to prevent endogenous thymidine synthesis, and 10 $\mu$Ci of $^{125}$IUdR (5-iodo-2′-deoxyuridine) (sp. act. $8.5\,mCi\,mg^{-1}$; Amersham/Searle Corp., Arlington Heights, IL). After washing with RPMI-1640 medium, cells were adjusted to the desired concentration and injected intravenously (i.v.) into DBA/2 mice (5–6 animals per group). Four hours later the radioactivity of lungs was measured in a well-type crystal scintillation counter (Packard Model 1510, Downers Grove, IL). The results are expressed as the geometric mean of the percentage recovery of injected radioactivity.

### *Evaluation of $^{125}IUdR$* in vivo *uptake by different organs for testing cell proliferation*

Lethally irradiated (800 R) DBA/2 mice (6–8 animals per group) challenged i.v. with FLC-V or FLC-Vt 2–4 h after irradiation were injected 4 days later with a single i.p. dose (25 $\mu$g per mouse) of FUdR to decrease the availability of endogenous thymidine. One hour later the mice were inoculated with 0.5 $\mu$Ci of $^{125}$IUdR in 0.25 ml of 0.85% NaCl solution. Spleens, livers and lungs were removed 3 h later and $^{125}$IUdR not incorporated into DNA was eluted by soaking the organs in 70% ethanol for 3 days (Bennett, 1972). The incorporation of radioactivity was measured in a gamma scintillation counter. The $^{125}$IUdR uptake was expressed as the geometric mean of the percentage of the isotope injected. (See also Hofer & Hughes, 1970.)

*Cytotoxicity assay*

The activity of natural killer (NK) cells was determined in a $^{51}$Cr release assay, as previously described (Herberman *et al.*, 1974). Briefly, graded numbers of effector spleen cells were suspended in RPMI-1640 medium containing 10% heat inactivated Newborn Calf Serum (Flow Laboratories, UK) and 2% L-glutamine, admixed with $10^4$ $^{51}$Cr-labelled target cells in *U*-shaped 96-well microtitre plates (Greiner, CA and Söhne, Nürtingen, West Germany) in quadruplicate, in a final volume of 0.2 ml. The plates were incubated for 4 h at 37°C in a 5% $CO_2$ incubator. At the end of the incubation the plates were centrifuged (800 *g* for 10 min) and the radioactivity in 0.1 ml of the supernatant was measured in a gamma-scintillation counter.

*Calculation of the percentage of specific lysis*

Experimental results were expressed as the percentage of specific lysis over spontaneous release and were calculated as follows:

$$\% \text{ specific lysis} = \frac{\text{c.p.m.}_{\text{t}} - \text{c.p.m.}_{\text{s}}}{\text{c.p.m.}_{\text{T}}} \times 100,$$

where c.p.m.$_t$ is the mean c.p.m. released in the presence of effector cells, c.p.m.$_s$ is the mean c.p.m. released spontaneously by target cells, c.p.m.$_T$ is the total amount of $^{51}$Cr incorporated into target cells.

*Calculation of lytic units*

Dose–response curves were obtained by plotting the percentages of specific $^{51}$Cr release and the effector:target (E/T) ratios. The best fit curve for this function was found to be logarithmic in accordance with previous reports (Tentori *et al.*, 1985).

A lytic unit, $LU_n$, was defined as the number of effector cells, extrapolated from the dose–response curve, required to achieve *n*% specific target cell lysis (Thorn & Herney, 1976). The amount of $LU_n$ per $10^6$ cells was calculated by dividing $10^6$ by the number of splenocytes corresponding to 1 $LU_n$.

*Statistical analysis*

As for $^{125}$IUdR uptake test and clearance of radiolabelled cells, the statistical analysis was performed according to Student's *t* test calculated on the logs of the original c.p.m. In the test for cell proliferation in lethally irradiated mice, the $^{125}$IUdR background (i.e. that of irradiated mice not subjected to lymphoma challenge) was substracted from the $^{125}$IUdR uptake of the test groups before calculations. For survival studies, the non-parametric Mann–Whitney *U* test was used.

Differences in cytolytic effects produced by NK effectors in various experimental conditions were evaluated taking into account the percent of specific cytotoxicity at all E/T ratios. Therefore *P* values were calculated using covariance analysis performed on the regression of the number of effector cells over the logs of the percentage of specific $^{51}$Cr release.

### Results

*Survival of DBA/2 mice injected i.v. with graded numbers of FLC-V or FLC-Vt*

Previous studies demonstrated marked differences in tumorigenicity of FLC-V and FLC-Vt after inoculation into syngeneic DBA/2 mice mainly by i.p. or s.c. routes (Belardelli *et al.*, 1984). Tumour titration carried out in DBA/2 hosts confirms that the same differences can be observed after i.v. inoculation of both FLC-V or FLC-Vt (Table I). All mice died after i.v. injection of $5 \times 10^6$ FLC-V and FLC-Vt, but the median survival time (MTS) was significantly higher in DBA/2 mice inoculated with FLC-Vt as compared with that of mice injected with FLC-V. When $10^6$ cells were used for tumour challenge no animal death was detected in mice inoculated with FLC-Vt, whereas all mice challenged with FLC-V died with generalized leukaemia within 17 (745) or 13 (3C18) days. In addition, mortality of mice recipient of FLC-V was 100% following inoculum of $10^5$ cells.

*Weakening of* in vivo *resistance to FLC-Vt growth by treatment with cyclophosphamide or sublethal irradiation*

To assess the potential role of host's immunity in the low tumorigenicity seen in normal syngeneic DBA/2 mice injected i.v. with FLC-Vt, $10^6$ *in vitro* passaged tumour cells were injected i.v. into mice suppressed by 400 R or by pretreatment with cyclophosphamide (200 mg kg$^{-1}$ i.p.). As shown in Table II, the incidence of lethal growth in immunosuppressed mice was 100%, whereas the majority of non-treated mice survived beyond the 60-day observation period.

**Table I** Survival of DBA/2 mice inoculated i.v. with FLC-V or FLC-Vt lines

| | | In vivo *passaged (FLC-V)* | | In vitro *passaged (FLC-Vt)* | |
|---|---|---|---|---|---|
| *Tumour cells* | *Dose* | *MST*[a] | *D/T*[b] | *MST* | *D/T* |
| FLC-745 | $5 \times 10^6$ | 11 | 11/11 | 22* | 11/11 |
| FLC-745 | $10^6$ | 12 | 6/6 | >60** | 0/6 |
| FLC-745 | $10^5$ | 15.5 | 6/6 | | n.d.[c] |
| FLC-3C18 | $5 \times 10^6$ | 8 | 11/11 | 24* | 7/7 |
| FLC-3C18 | $10^6$ | 10 | 5/5 | >60** | 0/6 |
| FLC-3C18 | $10^5$ | 13 | 5/5 | | n.d. |

[a]Median survival time (days); [b]dead over total mice tested; [c]n.d., not done.

*$P<0.02$; **$P<0.01$ according to Mann–Whitney *U* test, comparing the mortality data of mice injected with FLC-V with those of mice injected with FLC-Vt.

**Table II** Mortality of normal, cyclophosphamide-pretreated or sublethally irradiated DBA/2 mice (8-week old) after injection of $10^6$ FLC-Vt

| *Tumour cells* | *Pretreatment* | *MST*[a] | *D/T*[b] |
|---|---|---|---|
| FLC-745-Vt | – | >60 | 2/5 |
| FLC-745-Vt | cyclophosphamide[c] | 17* | 7/7 |
| FLC-745-Vt | 400 R[d] | 17* | 7/7 |
| FLC-3C18-Vt | – | >60 | 1/7 |
| FLC-3C18-Vt | cyclophosphamide | 22* | 7/7 |
| FLC-3C18-Vt | 400 R | 24* | 7/7 |

[a]Median survival time (days); [b]dead over total mice tested; [c]200 mg kg$^{-1}$ i.p. −2 days; [d]mice were exposed to 400 rads of gamma-rays 2 h before tumour injection.

*$P<0.02$ according to Mann–Whitney *U* test, comparing the mortality data of untreated mice with those of cyclophosphamide-treated or irradiated mice.

*Rapid* in vivo *elimination of* $^{125}$*IUdR-labelled FLC: age dependence and comparison between FLC-V and FLC-Vt*

Previous investigations pointed out that NR, measured as rapid clearance of prelabelled cells, is detectable against both FLC-Vt and FLC-V (Iorio *et al.*, 1986, 1989). In the present study a comparison between NR against FLC-V and FLC-Vt in DBA/2 mice of different ages was performed. Mice were injected i.v. with $10^4$ FLC-V or FLC-Vt, labelled with $^{125}$IUdR, and lung clearance capability, considered to be the most sensitive measure of NR (Riccardi *et al.*, 1980), was determined 4 h later. The results, illustrated in Figure 1, show that: (a) lung clearance of FLC-V or-Vt was not statistically different in infant mice (18–20 days old); (b) retained radioactivity in the lungs of adult mice (49 and 77 days old) was significantly lower in the animals inoculated with FLC-Vt as compared with that of mice injected with FLC-V; (c) the rate of clearance of leukaemia cells from adult mice was significantly higher than that observed in infant mice both for FLC-V and FLC-Vt.

*Influence of the treatment with cyclophosphamide or carrageenan on lung rapid clearance of FLC-V or FLC-Vt*

DBA/2 mice untreated or pretreated with cyclophosphamide or carrageenan were injected i.v. with $10^4$ labelled FLC-V or FLC-Vt. The results obtained, illustrated in Figure 2, show that: (a) pretreatment with cyclophosphamide, carrageenan or anti-Asialo GM-1 antiserum (see footnote of the figure) significantly impaired lung clearance of FLC-V or FLC-Vt; (b) significantly higher levels of radioactivity were found in the lungs of untreated or cyclophosphamide pretreated DBA/2 mice injected with FLC-V as compared with levels found in the lungs of mice injected with FLC-Vt; (c) on the contrary, clearance of FLC-V and FLC-Vt in mice depressed for NR by carrageenan pretreatment did not show substantial differences.

*Comparison between the growth of graded numbers of FLC-V or FLC-Vt in lethally irradiated mice*

Previous studies (Kawano *et al.*, 1986) demonstrated that the distribution and survival of cells in the early phase after inoculation did not necessarily correlate with the final fate of

**Figure 1** Lung rapid clearance of $10^4$ $^{125}$IUdR-labelled FLC-V (open columns) or FLC-Vt (filled columns) (745 or 3C18 clones) injected into syngeneic DBA/2 mice of different ages. The statistical analysis was performed according to Student's *t* test, comparing the logarithms of the recovery values. A, $P<0.01$ (FLC-V *vs* FLC-Vt); B, $P<0.05$ (FLC-V in infant (i.e. 18–20 days old) *vs* FLC-V in adult mice); C, $P<0.01$ (FLC-Vt in infant (i.e. 18–20 days old) *vs* FLC-Vt in adult mice).

**Figure 2** Lung rapid clearance of $2\times10^4$ $^{125}$IUdR-labelled FLC-V (open columns) or FLC-Vt (filled columns) (745 or 3C18 clones) injected i.v. into untreated or depressed for NR (cyclophosphamide (Cy 300 mg kg$^{-1}$ i.p. day $-1$) or carrageenan (CAR, 1 mg mouse$^{-1}$ day $-1$) pretreatment) syngeneic DBA/2 mice. The statistical analysis was performed according to Student's *t* test, comparing the logarithms of the recovery values. A, B or C, $P<0.01$; A, FLC-V *vs* FLC-Vt; B, FLC-V in untreated mice *vs* FLC-V in recipients pretreated with cyclophosphamide or carrageenan; C, FLC-Vt in untreated mice *vs* FLC-Vt in recipients pretreated with cyclophosphamide or carrageenan. Treatment of recipient mice with rabbit anti-Asialo GM-1 serum (Waco Chemicals, West Germany, 25 µl mouse$^{-1}$ i.v., 1 day before test) reduced leukaemia cell clearance in the lung by a factor of approximately 2 in the case of FLC-V and of 20 in the case of FLC-Vt. No substantial differences were found between 745 or 3C18 clones.

**Table III** Growth of graded numbers of FLC-V or FLC-Vt inoculated i.v. into lethally irradiated (750 R) DBA/2 mice (7 weeks old)

| | | % $^{125}$*IUdR uptake*[a] | |
|---|---|---|---|
| *Tumour cells* | *Dose* | *Spleen* | *Liver* |
| FLC-745-V | $5\times10^6$ | 3.46(3.24–3.69) | 1.35(1.22–1.49) |
| FLC-745-Vt | $5\times10^6$ | 0.04(0.03–0.05)* | 0.11(0.09–0.14)* |
| FLC-745-V | $2.5\times10^6$ | 1.41(1.31–1.53) | 0.53(0.51–0.56) |
| FLC-745-Vt | $2.5\times10^6$ | 0.05(0.04–0.06)* | 0.13(0.12–0.14)* |
| FLC-745-V | $10^6$ | 0.85(0.79–0.92) | 0.43(0.39–0.47) |
| FLC-745-Vt | $10^6$ | 0.03(0.02–0.04)* | 0.08(0.07–0.10)* |
| FLC-3C18-V | $5\times10^6$ | 4.42(3.28–5.95) | 1.56(1.09–2.24) |
| FLC-3C18-Vt | $5\times10^6$ | 0.05(0.04–0.06) | 0.13(0.11–0.15)* |
| FLC-3C18-V | $2.5\times10^6$ | 1.28(1.10–1.50) | 1.07(0.93–1.24) |
| FLC-3C18-Vt | $2.5\times10^6$ | 0.04(0.03–0.07)* | 0.08(0.07–0.09)* |
| FLC-3C18-V | $10^6$ | 0.25(0.19–0.34) | 0.85(0.73–0.98) |
| FLC-3C18-Vt | $10^6$ | 0.05(0.03–0.07)* | 0.09(0.08–0.11)* |

[a]Percentage of $^{125}$IUdR uptake (geometric mean) tested on day 4 after challenge. In parentheses the lower and upper limits of the mean within its standard error. The percentage of $^{125}$IUdR uptake in the lungs of mice injected with FLC-V or FLC-Vt was always less than 0.05%.

*$P<0.001$ according to Student's *t* test, comparing the values of percentage uptake found in mice injected with FLC-V with those obtained in mice injected with FLC-Vt.

organ colonization potential. For this reason we decided to investigate whether differences in sensitivity to NR measured as rapid lung clearance between FLC-V and FLC-Vt could be seen also in terms of growth in lethally irradiated mice (Iorio *et al.*, 1978). The results of the growth (4 days) of FLC-V or FLC-Vt injected i.v. into lethally irradiated DBA/2 mice are given in Table III. Using three different

**Table IV** Growth of FLC-Vt injected i.v. into lethally irradiated untreated, cyclophosphamide- or carrageenan-treated DBA/2 mice

| *Exp.* | *Tumour cells* | *Dose* | *Age (weeks)* | *Treatment* | *% $^{125}$IUdR uptake*[a] *Spleen* | *Liver* |
|---|---|---|---|---|---|---|
| 1 | FLC-745-Vt | $2\times10^6$ | 8 | – | 0.04(0.02–0.06) | 0.06(0.05–0.07) |
| | FLC-745-Vt | $2\times10^6$ | 8 | cyclophosphamide[b] | 0.05(0.03–0.07) | 0.05(0.05–0.06) |
| | FLC-745-Vt | $2\times10^6$ | 8 | carrageenan[c] | 0.28(0.21–0.37)** | 0.18(0.15–0.23)** |
| 2 | FLC-745-Vt | $2\times10^6$ | 13 | – | 0.15(0.10–0.21) | 0.08(0.07–0.09) |
| | FLC-745-Vt | $2\times10^6$ | 13 | cyclophosphamide | 0.42(0.34–0.52)* | 0.09(0.08–0.10) |
| | FLC-745-Vt | $2\times10^6$ | 13 | carrageenan | 0.50(0.35–0.72)* | 0.13(0.11–0.16) |
| 3 | FLC-3C18-Vt | $2\times10^6$ | 8 | – | 0.08(0.06–0.09) | 0.07(0.06–0.08) |
| | FLC-3C18-Vt | $2\times10^6$ | 8 | cyclophosphamide | 0.04(0.03–0.05) | 0.04(0.04–0.04) |
| 4 | FLC-3C18-Vt | $2\times10^6$ | 10 | – | 0.04(0.03–0.04) | 0.10(0.09–0.11) |
| | FLC-3C18-Vt | $2\times10^6$ | 10 | cyclophosphamide | 0.26(0.20–0.34)** | 0.13(0.12–0.14) |
| 5 | FLC-3C18-Vt | $5\times10^6$ | 8 | – | 0.03(0.02–0.04) | 0.06(0.05–0.07) |
| | FLC-3C18-Vt | $5\times10^6$ | 8 | carrageenan | 0.14(0.08–0.23)* | 0.18(0.16–0.21)** |

[a]Percentage of $^{125}$IUdR uptake (geometric mean) tested on day 4 after tumour challenge. In parentheses the lower and upper limits of the mean within its standard error; [b]300 mg kg$^{-1}$ i.p., day$^{-1}$; [c]1 mg mouse$^{-1}$ i.v., +3 h.
*$P<0.05$, **$P<0.01$ according to Student's *t* test, comparing the values of percentage uptake found in untreated mice with those obtained in mice treated with cyclophosphamide or carrageenan.

numbers of cells, a significantly higher proliferation was always observed in the spleen and liver of mice inoculated with FLC-V as compared with that found in mice injected with FLC-Vt. Essentially no tumour proliferation was observed in the lungs of mice inoculated with FLC-V or FLC-Vt (see footnote Table III) and in the liver and spleen of mice injected with FLC-Vt.

*Effect of treatment with cyclophosphamide or carrageenan on the growth of FLC-Vt injected i.v. into lethally irradiated DBA/2 mice*

In order to assess the role of natural immunity in impairing tumour proliferation in lethally irradiated mice injected with FLC-Vt, DBA/2 mice were immunosuppressed by treatment with cyclophosphamide or carrageenan, lethally irradiated and injected i.v. with FLC-Vt. The results of the growth of FLC-Vt, monitored by $^{125}$IUdR uptake 4 days later, are given in Table IV. Leukaemia cell proliferation was essentially absent in control and in cyclophosphamide-pretreated young (8 week old) DBA/2 mice injected i.v. with $2\times10^6$ FLC-745-Vt (exp. 1) or FLC-3C18-Vt (exp. 3). Significantly higher leukaemia cell proliferation was observed in the spleen of older DBA/2 mice (10–13 week old) pretreated with cyclophosphamide as compared with that in recipient controls (exp. 2 and 4). Treatment with carrageenan was more efficient and induced a significantly higher proliferation of FLC-Vt (both 745 and 3C18 clone) in the spleen and liver of young (exp. 1 and 5) or old (exp. 2) DBA/2 hosts as compared with that in untreated controls.

In vitro *NK activity against FLC-V or FLC-Vt*

Splenic NK activity of normal or Poly I:C stimulated DBA/2 mice was tested against FLC-V or FLC-Vt. In accordance with previous observations (Belardelli *et al.*, 1982*a*) FLC-V and FLC-Vt were resistant to NK-mediated lysis of spleen cells obtained from untreated DBA/2 mice (see footnote in Table V). On the other hand, a higher, but limited susceptibility of FLC-Vt as compared with that of FLC-V was observed when effector cells were collected from Poly I:C stimulated donors (Table V).

**Table V** Susceptibility of FLC clones to NK-mediated cytotoxicity of spleen cells collected from Poly I:C stimulated (5 mg kg$^{-1}$, day−1) DBA/2 mice

| *Group no.* | *Target cells*[a] | *$LU_5/10^6$*[b] *Mean (s.d.)* | *P*[c] |
|---|---|---|---|
| 1 | FLC-745-V | 0.2(0.03–1.5) | – |
| 2 | FLC-745-Vt | 16.6(14.5–19.0) | <0.01 |
| 3 | FLC-3C18-V | 5.7(3.9–8.3) | – |
| 4 | FLC-3C18-Vt | 11.3(8.8–14.6) | <0.01 |

[a]Effector:target ratios used: 100:1, 50:1, 25:1; [b]Lytic units 5% per $10^6$ effector cells. Data are expressed as mean (mean − standard deviation; mean + standard deviation). The splenic NK cytotoxic activity value of untreated DBA/2 mice to FLC-V and FLC-Vt never exceeded 3% of the mean; [c]*P*, probability, calculated as illustrated in **Materials and methods**, comparing groups 1 *vs* 2 and 3 *vs* 4.

### Discussion

The results of the present paper show that the scarcely oncogenic FLC-Vt clone is more susceptible to NR *in vivo* and *in vitro* as compared with the highly oncogenic FLC-V. This appears to be in line with the hypothesis that IFN could activate host-mediated mechanisms rather than directly acting on tumour cells (Gresser *et al.*, 1983).

A series of investigations has been undertaken in order to elucidate the mechanism of the indirect effect of IFN on the *in vivo* growth of tumour cells. Using models different from FLC, Kataoka *et al.* (1984) demonstrated the involvement of T-cell immunity, Uno *et al.* (1985) evidenced a positive contribution of macrophages, and Hanna (1980) and Hanna & Fidler (1980, 1981) observed that IFNs and IFN inducers could limit metastases in mice by stimulation of NK-cell system. However, other reports did not support the idea that host's immune mechanisms are involved in the indirect effect of IFN. Studies relative to the tumour model adopted in the present paper showed that IFN-induced FLC-V necrosis was not accompanied by host immunocyte infiltration (Belardelli *et al.*, 1982*b*). Therefore, it was proposed that IFN-dependent elimination of tumour cells could be mediated by various biochemical mechanisms such as changes in the physicochemical conditions within the peritoneal cavity. Moreover, Proietti *et al.* (1986), using $^{31}$P-nuclear resonance spectroscopy examinations, hypothesised that IFN could exert some effects on FLC metabolism via altered host microenvironment with subsequent tumour cell degeneration.

Investigations performed in our laboratory (Iorio *et al.*, 1986, 1989) examined the possibility that NR could be implicated in the indirect role played by IFN on FLC growth in mice. Host's immunity related to NR, measured *in vivo* as rapid clearance of prelabelled cells, was found to be present against FLC-V and FLC-Vt, independently from IFN-

sensitivity (Iorio *et al.*, 1986, 1989). The results shown in the present study were obtained by simultaneously measuring and comparing within the same experiment the following parameters concerning FLC-V and FLC-Vt: (a) mortality of syngeneic DBA/2 hosts following i.v. injection of the tumours; (b) specific organ retention of prelabelled cells at 4 h after i.v. inoculation; (c) growth of FLC in lethally irradiated mice; (d) susceptibility of the neoplastic cells to the effectors of *in vitro* measured NK activity.

The results of survival experiments (Table I) showed that FLC-V is more oncogenic than FLC-Vt when inoculated by the i.v. route, thus confirming the previous observations obtained injecting the same clones subcutaneously or intraperitoneally (Belardelli *et al.*, 1984). In addition the role of host's immunity was pointed out by the finding that resistance to FLC-Vt could be weakened by total-body irradiation or by pretreatment with an immunodepressive agent such as cyclophosphamide used at high dose (i.e. 200 mg $kg^{-1}$ i.p., Table II).

The studies performed to measure the possible involvement of NR *in vivo* in the low oncogenicity of FLC-Vt show significantly higher rates in FLC-Vt or FLC-V clearance in adult young mice as compared with that observed in infant recipients, immature for NR (Figure 1). Moreover, significantly higher amounts of radiolabelled cells were found in FLC-V injected young adult mice as compared with those detected in the hosts of the same age inoculated with FLC-Vt. No differences were present, instead, in infant non-immunocompetent mice. Depression of NR by pharmacological manipulation of the host (i.e. treatment with cyclophosphamide, carrageenan or anti-Asialo-GM-1 serum) induced a decline in the ability of mice to clear FLC-V or FLC-Vt (Figure 2). It is conceivable that NR-depressive agents act directly on NR-effector cells, including lung macrophages and NR-accessory cells involved in the upregulation of the natural immune function, possibly including IFN-gamma-producing T-cells (Friedman & Vogel, 1983).

The results of studies carried out measuring *in vivo* growth of FLC-V or FLC-Vt in lethally irradiated hosts are in accordance with data obtained measuring the rapid clearance of tumour cells. The proliferation of graded numbers of FLC-V was significantly higher than that observed for FLC-Vt (Table III). Actually no growth of FLC-Vt was observed in untreated lethally irradiated DBA/2 hosts, whereas significant proliferation occurred in mice pretreated with immunodepressive agents (Table IV).

Finally, the NK experiments *in vitro* confirmed that FLC-Vt is more susceptible than FLC-V to NK-mediated lysis. The observation that rapid *in vivo* clearance of FLC-Vt is markedly more efficient than *in vitro* killing by NK effector spleen cells, can be explained by at least two different hypotheses: (a) lung effector cells are more cytotoxic than splenocytes against FLC-Vt targets; (b) the kinetics of *in vivo* clearance of leukaemic cells results from a mechanism more complex than that underlying *in vitro* natural cell-mediated cytotoxicity. The data of increased susceptibility to NK-mediated lysis of FLC-Vt *vs* FLC-V are consistent with a number of studies reporting that tumour cells become less tumorigenic (Liu *et al.*, 1977; Morgan *et al.*, 1979; Beer *et al.*, 1983; Yamashina *et al.*, 1986) and more easily lysed *in vitro* by NK cells (Sendo *et al.*, 1975; Nunn *et al.*, 1977), following *in vitro* cultivation. This often seems to be due to increased immunogenicity presumably as a consequence of changes on the membrane surface (Liu *et al.*, 1977; Morgan *et al.*, 1979; Yamashina *et al.*, 1986). In fact, Amici *et al.* (1984), studying cell surface glycoproteins of FLC-V and FLC-Vt, observed a modified type and/or rate of glycosylation of membrane proteins possibly responsible of the differences in oncogenicity.

In conclusion, the results of the present investigation point to an important role of NR in the defence against Friend virus erythroleukaemia cells, although the mechanism underlying the role played by IFN has not been directly and definitely elucidated. In any case our data provide further support to the studies on host's surveillance in Friend virus-induced leukaemogenesis. Kumar *et al.* (1974) and Bennett *et al.* (1976) suggested that the natural effector cells active in haemopoietic graft rejection by lethally irradiated mice are similar to those involved in 'genetic resistance' to Friend virus leukaemia cells. Indeed, adult mice depleted of functional NK cell activity and of reactivity against haemopoietic histocompatibility (Hh) type antigens with the bone-seeking isotope $^{89}$Sr are deficient in their ability to suppress Friend virus erythroleukaemogenesis. Moreover, Eckner *et al.* (1987) found that treatment with anti-Asialo-GM1 serum, capable of depressing NK and Hh-type reactivity, elicited the rapid development of dormant leukaemia induced with replication-defective Friend polycythaemia-inducing spleens focus-forming virus ($SFFV_p$). Enhanced expression of Hh type antigens, target for marrow graft rejection, was observed by Rossi *et al.* (1970) and Cudkowicz *et al.* (1972) in DBA/2 spleen cells after *in vivo* infection with Friend leukaemia virus. Finally NR, measured as growth in lethally irradiated mice, was observed by Afifi *et al.* (1986) against FLD-3 erythroleukaemia, induced by Friend virus in BALB/c mice.

The work described in this paper was supported in part by grants from Consiglio Nazionale delle Ricerche, Rome, Italy, Progetto Finalizzato 'Oncologia', nos. 84.07577.44 and 87.02812.44.

### References

AFFABRIS, E., JEMMA, C. & ROSSI, G.B. (1982). Isolation of interferon-resistant variants of Friend erythroleukemia cells: effects of interferon and ouabain. *Virology*, **120**, 441.

AFIFI, M.S., BENNETT, M. & KUMAR, V. (1986). Natural immunity to grafts of FLD-3 erythroleukemia cells by irradiated mice. *Nat. Immun. Cell Growth Regul.*, **5**, 200.

AMICI, C., FERRANTINI, M., BENEDETTO, A., BELARDELLI, F. & GRESSER, I. (1984). Biologic and biochemical differences between *in vitro* and *in vivo* passaged Friend erythroleukemia cells. II. Changes in cell surface glycoproteins associated with a highly malignant phenotype. *Int. J. Cancer*, **34**, 397.

BEER, J.Z., BUDZICKA, E., NIEPOKOJEZYCKAK, E., ROSIEK, O.K., SZUMIEL, I. & WALIKA, M. (1983). Loss of tumorigenicity with simultaneous changes in radiosensitivity and photosensitivity during *in vitro* growth of L51178Y murine cells. *Cancer Res.*, **43**, 4736.

BELARDELLI, F., GRESSER, I., MAURY, C. & MAUNOURY, M.T. (1982*a*). Antitumor effects of interferon in mice injected with interferon-sensitive and interferon-resistant Friend leukemia cells. I. *Int. J. Cancer*, **30**, 813.

BELARDELLI, F., GRESSER, I., MAURY, C. & MAUNOURY, M.T. (1982*b*). Antitumor effects of interferon in mice injected with interferon-sensitive and interferon-resistant Friend leukemia cells. II. Role of host mechanisms. *Int. J. Cancer*, **30**, 821.

BELARDELLI, F., FERRANTINI, M., MAURY, C., SANTURBANO, L. & GRESSER, I. (1984). Biochemical differences between *in vitro* and *in vivo* passaged Friend erythroleukemia cells. I. Tumorigenicity and capacity to metastasize. *Int. J. Cancer*, **34**, 389.

BENNETT, M. (1972). Rejection of marrow allograft. Importance of H-2 homozygosity of donor cells. *Transplantation*, **14**, 289.

BENNETT, M., BAKER, E.E., EASTCOTT, J.W., KUMAR, V. & YONKOSKY, D. (1976). Selective elimination of marrow precursors with the bone seeking isotope 89Sr: implication for hemopoiesis, lymphopoiesis, viral leukemogenesis, and infection. *J. Reticuloendoth. Soc.*, **20**, 71.

CUDKOWICZ, G., ROSSI, G.B., HADDAD, J.R. & FRIEND, C. (1972). Hybrid resistance to parental DBA/2 grafts: independence from the H-2 locus. II. Studies with Friend virus induced leukemia cells. *J. Natl Cancer Inst.*, **48**, 997.

ECKNER, R.J., BENNETT, M., HETTRICK, K.L. & SEIDLER, C. (1987). Natural killer cell suppression of Friend virus-induced preleukemic hemopoietic stem cells. *J. Virol.*, **61**, 2631.

FRIEDMAN, R.M. & VOGEL, J. (1983). Interferons with special emphasis on the immune system. *Adv. Immunol.*, **34**, 97.

GRESSER, I., BELARDELLI, F., MAURY, C., MAUNOURY, M.T. & TOVEY, M.G. (1983). Injection of mice with antibody to interferon enhances the growth of transplantable murine tumours. *J. Exp. Med.*, **158**, 2095.

GRESSER, I., MAURY, C., WOOD ROW, D., MOSS, J. and 4 others (1988). Interferon treatment markedly inhibits the development of tumor metastases in the liver and spleen and increase survival time of mice after intravenous inoculation of Friend erythroleukemia cells. *Int. J. Cancer*, **41**, 135.

HANNA, N. (1980). Expression of metastatic potential of tumor cells in young mice is correlated with low levels of natural killer cell-mediated cytotoxicity. *Int. J. Cancer*, **26**, 675.

HANNA, N. & FIDLER, I.J. (1980). Role of natural killer cells in the destruction of circulating tumor emboli. *J. Natl Cancer Inst.*, **65**, 801.

HANNA, N. & FIDLER. I.J. (1981). Expression of metastatic potential of allogeneic and xenogeneic neoplasms in young nude mice. *Cancer Res.*, **41**, 438.

HERBERMAN, R.B., AOKI, T., NUNN, M.E. and 5 others (1974). Specificity of $^{51}$Cr-release cytotoxicity of lymphocytes immune to murine sarcoma virus. *J. Natl Cancer Inst.*, **53**, 1103.

HOFER, K.G. & HUGHES, L. (1970). Incorporation of iododeoxyuridine $^{125}$I into the DNA of L1210 leukemia cells during tumor development. *Cancer Res.*, **30**, 236.

IORIO, A.M., CAMPANILE, F., NERI, M., SPREAFICO, F., GOLDIN, A. & BONMASSAR, E. (1978). Inhibition of lymphoma growth in the spleen and liver of lethally irradiated mice. *J. Immunol.*, **120**, 1679.

IORIO. A., NERI, M., FEDERICO, M., ROSSI, G.B. & BONMASSAR, E. (1986). Natural resistance in mice against Friend leukemia cells. I. Studies with *in vitro* passaged interferon-sensitive and interferon-resistant cell clones. *Cell. Immunol.*, **98**, 230.

IORIO, A.M., NERI, M., ZEI, T., ROMEO, G., ROSSI, G.B. & BONMASSAR, E. (1989). Natural resistance in mice against Friend leukemia cells. II. Studies with *in vivo* passaged interferon sensitive and interferon-resistant cell clones. *Cell. Immunol.*, **118**, 425.

KATAOKA, T., OH-HASHI, F., SAKURAI, Y., USUKI, K. & IDA, N. (1984). Relative contribution of antiproliferative and host immunity-associated activity of mouse interferon in murine tumor therapy. *Cancer Res.*, **44**, 5661.

KAWANO, Y., TANIGUCHI, K., TOSHITANI, A. & NOMOTO, K. (1986). Synergistic defense system by cooperative natural effectors against metastases of B16 melanoma cells in H-2-associated control: different behavior of H-2$^+$ and H-2$^-$ cells in metastatic processes. *J. Immunol.*, **136**, 4729.

KUMAR, V., BENNETT, M. & ECKNER, R.J. (1974). Mechanism of genetic resistance to Friend leukemia virus in mice. I. Role of 89Sr-sensitive effector cells responsible for rejection of bone marrow allografts. *J. Exp. Med.*, **139**, 1093.

LIU, W.T., ROGERS, M.J., LAW, L.L. & CHANG, K.S.S. (1977). Properties of RBL-5 leukemia cells cultivated *in vitro*. *J. Natl Cancer Inst.*, **58**, 1661.

MORGAN, J.P., ENG, C.P., HEUCHEST, M.D. & KIRK, H.D. (1979). Loss of transplantability and induction of immunoprotection by mouse ascites tumor cells in tissue culture (34782). *Proc. Soc. Exp. Biol. Med.*, **134**, 305.

NUNN, M.E., HERBERMAN, R.B. & HOLDEN, H.T. (1977). Natural cell-mediated cytotoxicity in mice against non-lymphoid tumor cells and some normal cells. *Int. J. Cancer*, **20**, 381.

PROIETTI, E., CARPINELLI, G., DI VITO, M., BELARDELLI, F., GRESSER, I. & PODO, F. (1986). 31P-nuclear magnetic resonance analysis of interferon-induced alterations of phospholipid metabolites in interferon-sensitive and interferon-resistant Friend leukemia cell tumors in mice. *Cancer Res.*, **46**, 2849.

RICCARDI, C., SANTONI, A., BARLOZZARI, T., PUCCETTI, P. & HERBERMAN, R.B. (1980). *In vivo* natural reactivity of mice against tumor cells. *Int. J. Cancer*, **25**, 475.

ROSSI, G.B., CUDKOWICZ, G. & FRIEND, C. (1970). Transformation of spleen cells one day after infection of mice with Friend leukemia virus. *J. Exp. Med.*, **131**, 765.

SENDO, F., AOKI, T., BOYSE, E.A. & BUAFO, C.K. (1975). Natural occurrence of lymphocytes RLO showing cytotoxic activity to BALB/c radiation-induced leukemia cells. *J. Natl Cancer Inst.*, **55**, 603.

TENTORI, T., ALVINO, E., FUGGETTA, M.P. and 4 others (1985). Natural cell-mediated cytotoxicity: a micro assay suitable for clinical test. *Chemioterapia*, **4**, 471.

THORN, R.M. & HERNEY, C.S. (1976). Kinetic analysis of target cell destruction by effector T-cells. Delineation of parameters related to the frequency of killer cells. *J. Immunol.*, **117**, 2213.

UNO, K., SMIMIZU, S., IDO, M. and 5 others (1985). Direct and indirect effects of interferon on *in vivo* murine tumor cell growth. *Cancer Res.*, **45**, 1320.

YAMASHINA, K., OIKAWA, T., KASAI, M., NAIKI, M., CHIBA, I. & KOBAYASHI, H. (1986). Development of highly immunogenic variants of a rat fibrosarcoma line during *in vitro* cultivation. *Cancer Immunol. Immunother.*, **21**, 45.